# Advanced health biotechnologies in Thailand: redefining policy directions

**DOI:** 10.1186/1479-5876-11-1

**Published:** 2013-01-02

**Authors:** Román Pérez Velasco, Usa Chaikledkaew, Chaw Yin Myint, Roongnapa Khampang, Sripen Tantivess, Yot Teerawattananon

**Affiliations:** 1 Pharmaceutical Consultant, Bangkok, Thailand; 2 Health Intervention and Technology Assessment Program (HITAP), Ministry of Public Health, Tiwanon Rd, Muang, Nonthaburi, 11000, Thailand; 3 Social and Administrative Pharmacy Excellence Research (SAPER) Unit, Department of Pharmacy, Faculty of Pharmacy, Mahidol University, 447 Sri-Ayudthaya Road, Payathai, Ratchathevee, Bangkok, 10400, Thailand

**Keywords:** Advanced health biotechnologies, Advanced therapies, Pharmacogenomics, Stem cell therapy, Gene therapy, Tissue engineering therapy, Qualitative research, Biomedical research policy, Health policy, Thailand

## Abstract

**Background:**

Thailand faces a significant burden in terms of treating and managing degenerative and chronic diseases. Moreover, incidences of rare diseases are rising. Many of these—such as diabetes, cancer, and inherited inborn metabolic diseases—have no definite treatments or cure. Meanwhile, advanced health biotechnology has been found, in principle, to be an effective solution for these health problems.

**Methods:**

Qualitative approaches were employed to analyse the current situation and examine existing public policies related to advanced health biotechnologies in Thailand. The results of this analysis were then used to formulate policy recommendations.

**Results:**

Our research revealed that the system in Thailand in relation to advanced health biotechnologies is fragmented, with multiple unaddressed gaps, underfunding of research and development (R&D), and a lack of incentives for the private sector. In addition, there are no clear definitions of advanced health biotechnologies, and coverage pathways are absent. Meanwhile, false advertising and misinformation are prevalent, with no responsible bodies to actively and effectively provide appropriate information and education (I&E). The establishment of a specialised institution to fill the gaps in this area is warranted.

**Conclusion:**

The development and implementation of a comprehensive national strategic plan related to advanced health biotechnologies, greater investment in R&D and I&E for all stakeholders, collaboration among agencies, harmonisation of reimbursement across public health schemes, and provision of targeted I&E are specifically recommended.

## Background

Although Thailand has been classified as a technology-recipient country [1], significant research and development (R&D) in several areas of biomedicine has been undertaken in recent years [2]. A number of excellence centres for biomedical research have been established in universities and other institutions, some of which work on advanced health biotechnologies. Furthermore, a number of private companies for stem cell research are operating in the country [3-8].

Prior to 2009, stem cell therapy interventions in clinical practice were entirely unregulated in Thailand. This led to many incidents of exaggerated and false claims and a number of cases of misconduct among clinicians offering stem cell therapy, which prompted increased worldwide attention on these issues [9-12]. In Thailand, stem cell therapy was provided by a number of institutions and individuals before sufficient research and development had been undertaken to ensure the safety of the procedure.

Despite the fact that there is significant regulation of stem cell research and unproven treatment is now forbidden, there continue to be breaches in the law. However, many positive advances in the field of pharmacogenomic/pharmacogenetic (PGx) testing have been made in recent years, with some testing already taking place in public hospitals [13]. At the same time, however, little attention has been paid to areas such as tissue engineering and gene therapy, which have advanced more slowly as a result.

To plan more effectively for the future, the Thai National Science, Technology and Innovation Policy Office commissioned the Health Intervention and Technology Assessment Program (HITAP), a research arm of the Health Ministry, to conduct research for the development of advanced health biotechnologies in Thailand. This paper provides an assessment of the current situation in Thailand, including a review of relevant public policy. Based on this review and analysis, we make a number of policy recommendations.

## Methods

A review of existing literature on advanced health biotechnologies in Thailand was undertaken using relevant bibliographic databases, search engines, and websites. The data was then organised into a conceptual framework. For benchmarking purposes, research was also conducted for regions such as the United States and Europe. This literature review was complemented by information garnered during four focus group discussions involving a number of relevant stakeholders, including Thai researchers, administrators of research institutes, policymakers, regulators, patient representatives, and physicians. These took place between October and December 2011 in the premises of HITAP. Expert opinions and data on the current situation and existing policies were also collected following attendance at an external consultation, convened by the National Science and Technology Development Agency (NSTDA). Two case studies on facilitators and barriers to the adoption and diffusion of advanced health biotechnologies were developed, and the findings from these also informed the study. Finally, two consultative meetings were held in HITAP in June 2011 and March 2012 to gather additional information, verify the initial results, and fine-tune the recommendations. Finally, triangulation was applied to verify the findings and recommendations. More detail on the research methods, including details of the relevant materials and definitions can be found in the full report, which is available upon registration at http://www.hitap.net/en/research/10664.

### Review of the situation and public policies

#### Research and development

Our research revealed that, while a policy document on advanced health biotechnologies has been developed by the NSTDA, [14] it fails to provide clear policy directions for undertaking research in this area. However, this finding is disputed by the key informant of the medical cluster of the NSTDA, which is responsible for R&D, technology transfer, human resources, and infrastructure development associated with science and technology. Nevertheless, as a result of the complex management structures that are in place at the NSTDA (each cluster has its own director), it is unclear how the policy outlined in the paper is actually implemented. This lack of a clear national policy and recommendations on research in advanced health biotechnologies has meant that many R&D activities are conducted outside of the scope of the National Research Council (NRC) and NSTDA. There is also very little communication between the relevant national bodies on which areas of advanced health biotechnologies in Thailand are supported.

It is recognised that large pharmaceutical firms may be hesitant to conduct R&D on advanced health biotechnologies for a number of reasons, including uncertainty surrounding the potential benefits and risks of these technologies, high production costs, strict regulations, and logistical difficulties [15]. Moreover, to overcome this, it may be necessary to develop innovative financial mechanisms for R&D and public-private partnerships (PPPs) in this field [16-20]. However, with no clear national policy in place, the benefits of such partnerships might not be maximised or might even result in serving mainly the commercial interests of those involved.

Owing to the lack of uniformity on international ethical standards in biomedical R&D, very little attention on this has been paid in Thailand. The system for the ethical approval of medical research has improved very little in four decades. One recent survey found that many of these ethical committees have poor capacity, lacking codes of conduct and proper regulations to avoid conflicts of interest [21]. In the area of advanced health biotechnologies, the situation may be worse even than in conventional biomedical research because of the rapid progress of scientific knowledge, involvement of human donors, and disclosure of close relatives’ genetic information, *inter alia*[22,23]. As a result, researchers have tended to conduct R&D without applying for ethical clearance. Moreover, even when ethical clearance is sought, it is frequently given routinely, with little or no analysis of the ethical implications of the research.

There have also been several cases where advanced health biotechnologies were applied in ways that violated public trust [24-26]; as a consequence, the Medical Council (MC), which is the medical professional regulatory body, has drafted stricter regulations that explicitly cover all stem cell research and practice conducted by medical doctors (except for bone marrow transplantation, which is viewed as a conventional procedure). Under these new regulations, all physicians wishing to undertake research on stem cell therapy must obtain both scientific and ethical approval from the MC. However, Thai experts suggest that many scientists regard this regulation as an obstacle to advancement in this field of research. At the same time, it appears that these regulations are not fully enforced, perhaps because of inadequate regulator capacity and the potential for conflict of interest [27]. This echoes a case in the US, where the California Institute for Regenerative Medicine (CIRM)—the funding agency with the largest amount of public funds in the state—had on its board a number of experts from leading research organisations who were responsible for approving grant applications, including those of their own institutes and their competitors [28].

Despite this, the CIRM funding approach has a number of notable benefits. Significantly, it implements a system where more stable funding of research is guaranteed by the provision of grants over several years, rather than on a year-by-year basis (which had been the case in previous years) [28]. This year-by-year allocation system of the limited government budget is the current practice among almost all Thai research granters, including the NRC and NSTDA. This system hampers the progress of research, as suggested by the Tufts Center for the Study of Drug Development, which estimates that one advanced health biotechnology product requires an average of 8 years at a cost of USD 1.2 billion, compared to the average period of seven years and cost of USD 800 million for one conventional medical product [28].

#### Authorisation

Many advanced health biotechnologies do not fall clearly into the classic categories used by many regulatory agencies. In Thailand, the Thai Food and Drug Administration (TFDA), Department of Medical Sciences (DMSc), and MC share responsibility for the authorisation of medical products, medical and laboratory practice, and related advertising. These organisations do not have a clear and uniform way of defining and classifying advanced health biotechnologies. In contrast, in Europe a consensus was reached in 2007, in the form of the Advanced Therapies legal and regulatory framework (Regulation (EC) No 1394/2007). In addition, the US Food and Drug Administration (FDA) has also developed definitions for regenerative medicine products (neither framework covers PGx tests, which fall under a different classification). Having clear definitions and classification is very important, as regulators can use them to set standards of information requirements for authorisation, reimbursement, and post-authorisation activities.

Evidence suggests that, in Thailand, there is both insufficient demand for regulation of advanced health biotechnologies and inadequate capacity to implement this regulation should the demand arise. This results from a number of factors. First, there is a lack of clarity regarding which body would be responsible for regulating these kinds of technologies. For instance, the TFDA oversees only pharmaceutical products (including biologics) and medical devices, unlike the European Medicines Agency (EMA) and the FDA, which have specific expertise in these kinds of technologies. Furthermore, private biotechnology companies are not pushing for approval of their products because this may not suit their business needs; for example, many PGx tests are locally produced by healthcare facilities, which renders them outside the remit of the TFDA’s regulations. In addition, the Thai market for advanced health biotechnologies is relatively small and, as such, private biotechnology manufacturers do not prioritise marketing in Thailand.

#### Post-authorisation

We have classified post-authorisation regulatory activities into the following four areas:

##### Post-marketing surveillance

In the US, the FDA plays a major role in post-marketing surveillance of advanced health biotechnologies, including cell therapy, tissue engineering, and other regenerative medicines [29]. In Europe, the EMA is responsible for product surveillance, but it varies in the case of monitoring of services, which is conducted by either national drug regulatory agencies or health care quality inspectorates [30]. Given that there is no clear definition or classification of advanced health biotechnologies in Thailand, we predict that ensuring safety, effectiveness, and quality of these technologies after approval is likely to be a challenge. Recently, the MC prohibited any stem cell therapies except bone marrow transplantation. This should only be regarded as a short-term solution to ensure the safety, effectiveness and quality of stem cell treatment; moreover, this regulation does not extend to surveillance after approval, which means that many therapies continue to be conducted without sufficient regulation or monitoring.

##### Quality assurance of laboratories in the service sector

There is no national authority in place in Thailand to regulate laboratory practice and quality related to advanced health biotechnologies—although the DMSc is responsible for Good Laboratory Practice (GLP) compliance monitoring. The DMSc is also increasing the capacity of its 15 laboratories across the country in order to perform a number of PGx tests, mainly focusing on preventing severe adverse reactions from anti-retroviral therapy and epilepsy treatment. It is unlikely that the DMSc will support the private laboratories in their attempt to build their PGx testing capacity. In addition, the Department has no plan to widen its scope to support lab activities related to regenerative therapies [31].

##### Quality assurance of medical services

The quality assurance system of medical services in Thailand is passive; that is, the MC only comes into play when complaints are made by patients. The MC investigates and punishes medical practitioners if their practice is substandard or negligent, or if they make exaggerated claims or undertake false advertising, but only after a report or complaint is made. Patients can also file a lawsuit in the civil and criminal courts. In addition, the MC controls the quality of services by defining those who are permitted to provide various services. This means that only those who are licensed as physicians can prescribe and perform medical care. At present, because there is no individual or body specifically tasked with setting standards of care for advanced health biotechnologies, undertaking quality control of medical services in this area is certain to be a challenge.

##### Promotion/advertisement regulation

In Europe, the advertising of regenerative medicine services has been prohibited by the European Commission, while the EMA regulates the advertising of advanced therapy products.

The US allows the advertising of prescription-only drugs, and, in principle, this should extend to approved advanced health biotechnology products. However, despite the FDA’s considerable capacity, problems are still encountered in terms of regulating advertisement and sales for approved advanced health biotechnology products. In particular, there are calls to restrict direct-to-consumer (DTC) genetic test advertisements and sales. In Thailand, the DTC sale of prescription-only products is prohibited by law, and the legal regulation of medical service advertising is supervised by the Bureau of Sanatorium and Art of Healing. Despite this, there are still many public advertisements for advanced health biotechnologies, almost all of which make exaggerated claims [25,32,33].

#### Reimbursement and related service provision models

The reimbursement of products related to advanced health biotechnologies is challenging even in the US and Europe. The assessment of safety, efficacy/effectiveness, acceptability, and other social consequences is difficult and varies depending on context, due to differences in patient and clinician behaviour and delayed health outcomes, among others. Moreover, the information available for reimbursement of advanced health biotechnology products is often inadequate when compared to that available for conventional treatment. The third party payer is often pressured to approve these technologies because of the lack of available alternatives. Thus, a number of scholars have recommended that those payers that are faced with a promising advanced health biotechnology that may improve patient safety and outcomes, but which still has significant uncertainty associated with it, introduce coverage with evidence development (CED) [34,35]. For example, in 2009, the US Center for Medicare and Medicaid Services initiated CED for warfarin PGx testing, given the clinical promise but inconclusiveness or contrary results that had emerged on the back of the few small randomised controlled trials (RCTs) that had been conducted at the time [35].

The service and financing models that are currently available in Thailand were designed for conventional medical technologies. Commonly, public providers buy medicines and medical devices (including medical supplies) from the private sector and manage them themselves, which can be seen as a pure purchaser-supplier relationship. The National Health Security Office (NHSO), which manages the Universal Health Coverage Scheme—the largest of the three public health plans in Thailand, also buys commodities at the central level and allocates them to local public providers. The latter business model is believed to increase system efficiency because the buyer has more price negotiation power, although this may come at the expense of future market competition and the providers’ autonomy [36]. In recent years, the NHSO has started to buy services from private companies, for example, cataract surgery, renal dialysis, and heart surgery. In addition, some public hospitals lease public space out to private companies to develop and operate service delivery units (SDUs), such as medical scanning or renal dialysis units; these arrangements operate under informal contracts [37]. This does not comply with government regulations and creates difficulties [38]. For instance, one study has indicated that most public hospitals do not have access to sufficient information and do not exert much power when conducting negotiations with private companies. As a result of this misconduct, these privately-run SDUs are unevenly introduced across regions, sometimes resulting in the overprescribing of services [37].

We believe that, if advanced health biotechnologies—and regenerative medicine in particular— are to be made available under public health plans, their delivery model is likely to take a similar form to that of the privately-run SDUs; this is because, while the technological know-how will belong to private sector entities, these entities will not be able to operate these services completely on their own. As a consequence, new models need to be developed for PPPs in the health sector in Thailand that can be tailored specifically tor advanced health biotechnologies.

#### Information and education

It is widely recognised that provision of information and education (I&E) for all stakeholders is critical if society is to maximise the benefits from advanced health biotechnologies. For instance, most clinicians in the US and Europe have little knowledge on genomic medicine or the benefits and risks of specific PGx tests. Moreover, many of them are concerned about the consequences of their inadequate knowledge and how this could potentially lead to mistakes and even liability [39]. This appears to delay the translation of advanced health biotechnologies into clinical practice.

In most countries, including Thailand, related research organisations and networks provide public I&E; however, given the limited capacity and the potential for conflict of interest, society should not rely only on these sources. In the US, the National Human Genomics Research Institute (NHGRI) established the Education and Community Involvement Branch (ECIB), a body specifically responsible for public education on genomics-related issues.

Similarly, many non-governmental organisations (NGOs) and patient advocacy groups have in place key policies to disseminate I&E as part of their strategy to garner public support for advanced health biotechnologies. Evidence from the US and EU suggests that, while NGO provision of I&E can be very effective, it can also create misunderstandings. In Thailand *ad hoc* public I&E sharing was conducted by the TFDA at the time when stem cell banks became a public interest. Currently, no public body actively provides I&E on advanced health biotechnologies to the public.

**Figure 1:**
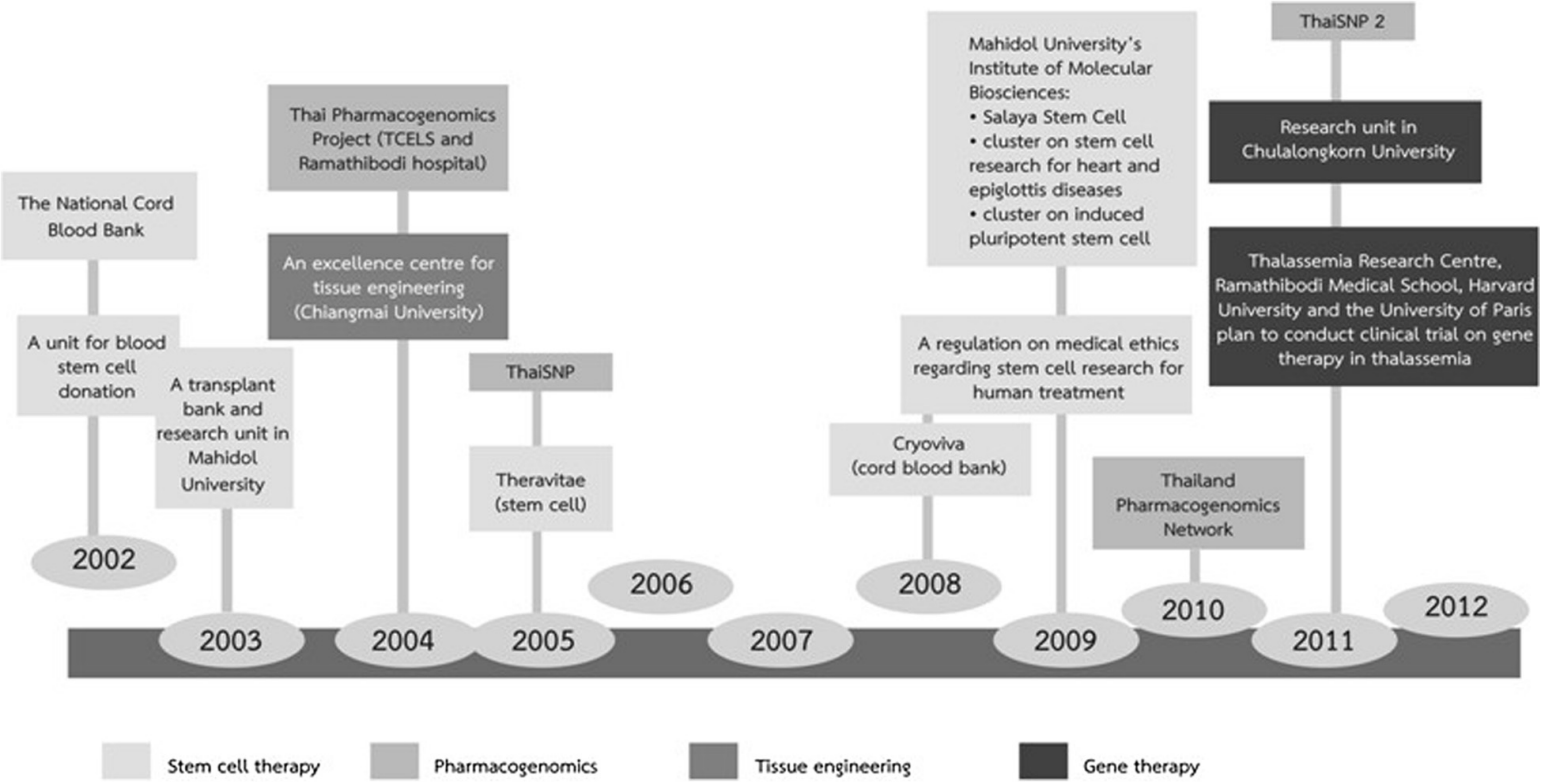
Milestones on the development of advanced health biotechnologies in Thailand during 2002–2012.

In Thailand, there are currently no effective programs to help health professionals update their knowledge on advanced health biotechnologies. Moreover, there is no license revalidation policy for health professionals, despite an attempt a few years ago to put one in place. In the US, the NHGRI is planning to recommend to professional associations that they integrate genomic medicine education into curricula and revalidation processes [40]. Approximately 30,000 doctors currently practice medicine in Thailand, and every year 1,000 newly graduated physicians enter the profession [41]. The majority of the workforce graduated before the application of these biotechnologies came into clinical practice. If more advanced health biotechnologies are approved for the market, it may be necessary to better regulate the provision of I&E pertaining advanced health biotechnologies by sales representatives to Thai physicians and other health professionals because of its importance [42,43]. Alternatively, or in addition, it may be useful for private companies who are involved in these technologies to invest in professional I&E rather than public. (Figure 1 provides a timeline of key events in the area in Thailand).

### Establishment of a specialised institute for advanced health biotechnologies

Having identified a number of significant gaps in the current system involving advanced health biotechnologies in Thailand, and mindful of the lessons learnt from the EU and US that are outlined above, we identified the need to establish an organisation focusing on advanced health biotechnologies in Thailand (sharing some characteristics with the Andalusian Initiative for Advanced Therapies [18,19] or the CIRM [28]). Such an organisation could be entitled the ‘Advanced Health Biotechnologies Institute’ (‘AHBI’) and would be tasked with overseeing the technologies under the scope of our study as well as other advanced health biotechnologies, such as nanomedicines. It should be an autonomous institute established outside the umbrella of the Ministry of Public Health and NSTDA of the Ministry of Science and Technology, because of the complex factors and implications involved with regulating advanced health biotechnologies, including environmental health, animal health, human health, and science characteristics. This is to avoid political conflict between the two Ministries, and is also aligned with the establishment of the National Research Council, under the Office of the Prime Minister rather than any particular Ministry.

The proposed responsibilities of this institute are as follows:

To provide national policy directions regarding R&D and related infrastructure, clinical application, I&E, and capacity strengthening, with reference to all stakeholders.

To provide research funding (including setting priorities for research and developing human resources).

To provide ethical/scientific approval for research.

To issue guidelines on good practice and clinical practice.

To provide public I&E and ensure that the quality of information offered by other public organisations is of the requisite standard.

To create a certification system, similar to the HONCode system, to control the quality of information published in websites, leaflets, magazines, and other media. This information should then be collated into a public database, which can serve as a resource for the public (including healthcare professionals) to access validated data on advanced health biotechnologies [44,45].

To cooperate with the school consortia of health professions for formal education, and the Royal Colleges to plan for and develop strategies relating to continuing education for health professionals after formal education.

To strengthen existing regulatory bodies (Department of Health Service Support, TFDA, Bureau of Sanatorium and Art of Healing, MC), to provide support for approval and post-approval activities, and cooperate with the Consumers’ Protection Office, TFDA and MC, in terms of advertisements and complaints.

### Particular recommendations for five key areas in Thailand

#### Research and development

It is essential that a national policy regarding R&D for advanced health biotechnologies in Thailand be established, including a prioritisation framework and scope. This national policy would be introduced not only by the ‘AHBI’ but also by other public fund-holders, such as the NRC and the NSTDA. To better respond to the rapid progress of the field, we recommend that the national strategic plans be five-yearly, with an option of rolling revision if needed. The plan should be developed under the ‘AHBI’ by a multi-sectoral team, including health professionals, scientists, policy researchers, patient groups, and the private sector. The national plan should include clear targets, and evaluation of its impacts in terms of population health, innovation, and national economics and competitiveness is also needed.

It is anticipated that the R&D market for advanced health biotechnologies will gradually develop; it is also likely that this market will be monopolised by one or a few firms. This may differ from the market in advanced economies because Thailand has a relatively small service market and limited human resources for R&D. The government should have strategies in place to avoid monopolies by private manufacturers or providers. Starting from the R&D process, the ‘AHBI’ should facilitate PPPs in an appropriate manner; particular care should be taken to avoid over-reliance on a single group of scientists or one private firm.

As previously discussed, as there is unlikely to be sufficient incentive for the private sector to invest in the R&D of advanced health biotechnologies as opposed to conventional health technologies, we recommend that the government allocate more resources to this field, to ensure that the public can take advantage of the benefits it offers. In addition, funding should be provided in a long-term, collaborative way, rather than on a year-by-year basis, as evidence suggests that it takes at least eight years to develop a final advanced health biotechnologies product [29]. This justification has informed funding policy in Europe and the US, as is evident from the approach of the CIRM and others. Furthermore, ethical standards for R&D must be clearly set by the ‘AHBI’ in order to avoid research misconduct. To this end, the ‘AHBI’ first needs to strengthen the capacity of the medical ethics committees through training. Secondly, for projects that are controversial or pose high risk of harm, the ‘AHBI’ should be ultimately responsible for appraising them [46]. Lastly, the ‘AHBI’ should open channels for complaints against suspected violation of research ethical standards.

#### Authorisation

We anticipate regulatory gaps and stress the need for collaboration among regulatory bodies to address these shortcomings, which include a lack of common definitions/classifications used by all regulators. Therefore, we recommend that the ‘AHBI’ work in conjunction with all stakeholders to establish agreed definitions/classifications of advanced health biotechnologies in Thailand. This can be done after conducting a review of experiences in the US, EU, and other Asian countries that are active in this field.

Second, regarding the approval of products, we recommend that the TFDA expand its current limited capacity to ensure the appropriate authorisation of advanced health biotechnologies. Currently, there is a gap in that there is no legal authority to approve individual health services (e.g., stem cell treatment). Although Royal Colleges in Thailand do play a role in setting standard clinical practice, this is informal. We recommend that the Bureau of Sanatorium and Art of Healing, in collaboration with the Royal Colleges, approves the use of individual advanced health biotechnology services, because of the uncertainties of safety and effectiveness, and the social and ethical implications.

In addition, the criteria for the authorisation of products and individual services should be harmonised using a modified framework for priority setting, as described above. Moreover, the developed framework should fully engage all relevant stakeholders (e.g., insurance managers, ethicists, HTA researchers, religious leaders, NGO representatives, etc.). The ‘AHBI’ should also play a consultation role for all regulatory bodies regarding the authorisation of advanced health biotechnologies. Another shortfall that needs to be addressed is the lack of capacity among regulators. We propose that the ‘AHBI’ invest in the capacity strengthening of regulators across the system, to ensure that there is uniformity across national policy and that, within networks, all knowledge and skills that are offered are relevant and up to date.

#### Post-authorisation

We expect that post-authorisation activities will be critical in the field of advanced health biotechnology because, as discussed, there is, as yet, no established requirement on evidence for authorisation; this means that many randomised controlled trials, if available, are likely to be so small that they cannot confirm long-term safety and effectiveness. Therefore, we offer the following recommendations for four elements of post-authorisation:

##### Post-marketing surveillance/withdrawal

The implementation of a stronger surveillance system, one which extends to patient registries, is necessary. So too is a risk management and a long-term monitoring and reporting system, which should be developed and maintained by manufacturers, working closely with health providers. Guidelines provided by the ‘AHBI’ should be followed by manufacturers (including hospitals), and those who do not comply should be held liable.

##### Quality assurance of laboratories in the service sector

Reinforcement of good practice guidelines should fall under the responsibility of the DMSc, and capacity strengthening should be provided by the ‘AHBI’. Quality assurance and the inspection of laboratories in the service sector should be conducted by the DMSc in collaboration with the TFDA.

##### Quality assurance of medical services

The ‘AHBI’ and the Royal Colleges should set the medical standards for those well-established advanced health biotechnologies that already have proven safe and effective. This will not only ensure quality of practice, but will also promote their use. In light of the fact that national standards related to conventional medical interventions were often established once these interventions had been accepted for reimbursement (because payers want to estimate costs and develop systems for monitoring and evaluation), we recommend that the standards for advanced health biotechnologies be set at the early stage of the introduction of these advanced health biotechnologies.

##### Promotion/advertising regulation

The ‘AHBI’ should implement a system similar to the HONCode system to certify the quality of information published in websites, leaflets, magazines, and other media. This information should then be collated into a public database, which can serve as a resource for the public (including healthcare professionals) to access validated data on advanced health biotechnologies. This is to complement the TFDA’s activities.

#### Reimbursement

Because many advanced health biotechnologies are likely to be expensive and will need close monitoring and evaluation for cohort patients, we strongly suggest that the reimbursement of these technologies be harmonised across public health plans in Thailand to facilitate the monitoring of safety and effectiveness. CED should be introduced for selected advanced health biotechnologies whose safety and effectiveness is as yet not fully proven. This also facilitates access to interventions for patients in need (complying with restricted criteria from both the TFDA and insurance managers), while collecting more evidence on effectiveness and safety.

Since Thailand has three major public health schemes managed by different bodies (i.e., NHSO, the Comptroller General’s Department, and the Social Security Office), we recommend that the coverage decision-making body be co-chaired by representatives of the three health schemes, with technical support provided by the ‘AHBI’, relevant professional associations, and HTA agencies. We recommend that some relevant indicators from a European framework be adopted (e.g., safety; knowledge/education; broader health impacts; and social, ethical, legal, and organisational aspects) [47]. In addition, there should be more collaboration between regulators, HTA agencies, and insurance managers in relation to information exchange and post-marketing surveillance, including CED.

For those products that involve a service, such as a medical/surgical procedure or diagnosis offered by the private sector in public hospitals (and we believe that most regenerative medicine technologies will take this form), the development of new purchasing models between public health insurance plans and private companies is needed, to ensure both effective administration and the equitable distribution of advanced health biotechnology services across the country. In addition, revision of the public procurement law is a necessary prerequisite because the current regulation does not allow private companies to provide clinical services within public health facilities.

#### Information and education

The ‘AHBI’ should be the organisation responsible for the provision of I&E for all stakeholders. To maximise the benefits that advanced health biotechnologies can offer society, the ‘AHBI’ should classify I&E activities for advanced health biotechnologies into three levels, and target stakeholders accordingly (Table 1).

**Table 1:** **Information and education targets according to level of evidence (partly based on**[48]**)**

	**Level 1 Well-established safety and effectiveness**	**Level 2 Established safety and efficacy, promising effectiveness**	**Level 3 Clear evidence on harm or disutility**
**Regulators/payers**	**✓**		**✓**
**Health professionals**	**✓**	**✓**	**✓**
**Research funders/ researchers**		**✓**	
**Media**			**✓**
**Public**	**✓**		**✓**

Note: in the case of PGx tests, efficacy and effectiveness should be replaced with validity and utility, respectively.

## Conclusion

In this paper, we outline a set of recommendations that aim to address the multiple gaps and weaknesses concerning advanced health biotechnologies in Thailand, ranging from underfunding of research to regulatory deficits. We conclude that the establishment of a specialised institute to fill the gaps in this area may represent the most practical approach to tackle the existing deficiencies in the Thai setting. Under a uniform national strategic plan, the government should also invest more in R&D and provide targeted I&E for all stakeholders in this area. All regulators would also need to work to gether with insurance providers and other stakeholders to ensure the safety, effectiveness and quality of advanced health biotechnologies. Moreover, the reimbursement of advanced health biotechnologies should be harmonised across public health plans in the country, and access to new technologies should be provided to restricted patient groups, if any impact on population health is to be realised.

## Competing interests

The authors declare that they have no competing interests.

## Authors’ contributions

RPV coordinated the project, reviewed the European situation, participated in the planning and implementation of the overall research, and contributed to the analysis and the writing of the draft. CYM reviewed the US situation, participated in the planning and implementation of the overall research, contributed to the analysis and the writing of the draft. UC, RK, and ST reviewed the Thai situation, analysed the focus group data, participated in the planning and implementation of the overall research, and contributed to the analysis and the writing of the draft; YT supervised the project, moderated the focus groups and consultations, and contributed to the analysis and the writing of the draft. All authors read and approved the final manuscript.

## Authors’ information

Román Pérez Velasco studied Pharmacy at the University of Granada, Spain, and earned his master’s in Clinical Pharmacy, International Practice and Policy at the School of Pharmacy, University College London, UK. He works as a consultant in the field of pharmaceutical policy and regulation.

Usa Chaikledkaew is a pharmacist; she earned her master’s degree in Economics and a PhD in Pharmaceutical Economics and Policy at the University of Southern California, US. She is an Assistant Professor and Deputy Dean for International Relations at the School of Pharmacy, Mahidol University, Thailand. Since 2006, she has been a research consultant. She is one of the founders of HITAP.

Chaw Yin Myint is a physician; she earned her master’s in Public Health at Mahidol University. She has experience in the implementation of public health programs and has worked in health technology assessment for two years.

Roongnapa Khampang earned her BSc in Health Sciences at Maastricht University, the Netherlands. She is currently pursuing an MSc in Epidemiology at Prince of Songkla University, Thailand.

Sripen Tantivess is a pharmacist; she earned her PhD in Public Health and Health Policy at the London School of Hygiene and Tropical Medicine in 2006. She is a founding member and senior researcher at HITAP.

Yot Teerawattananon is a leader and founder of HITAP. He previously served as a medical doctor and director of Pong Hospital in Northern Thailand. He earned his PhD in Health Economics in the UK in 2006.
